# Factors influencing quality of life in multiple sclerosis patients in Northwest Ethiopia: a multicentre cross-sectional study

**DOI:** 10.1080/07853890.2026.2700804

**Published:** 2026-07-13

**Authors:** Assefa Kebad Mengesha, Alemante Tafese Beyna, Demis Getachew, Liknaw Workie Limenh, Tewodros Ayalew Tessema, Habtamu Semagne Ayele

**Affiliations:** ^a^ Department of Pharmacology, School of Pharmacy, College of Medicine and Health Sciences, University of Gondar, Gondar, Ethiopia; ^b^ Department of Pharmaceutics, School of Pharmacy, College of Medicine and Health Sciences, University of Gondar, Gondar, Ethiopia

**Keywords:** Multiple sclerosis, quality of life, disease severity, treatment adherence, Northwest Ethiopia

## Abstract

**Background:**

Multiple sclerosis (MS) significantly affects patients’ quality of life (QoL), particularly in resource-limited settings. This multicentre study aimed to identify factors influencing QoL among MS patients in Northwest Ethiopia, where data are scarce.

**Methods:**

A cross-sectional study was conducted from January to June 2024 among 427 MS patients (≥18 years, diagnosed using McDonald criteria) attending three referral hospitals (University of Gondar, Debre Tabor, Felege Hiwot). Stratified sampling ensured representation across disease severity. Sociodemographic, clinical, psychological and behavioural data were collected. QoL was measured using the MSQOL-54 questionnaire. Multiple regression identified predictors of QoL, expressed as β coefficients and *p* values. Treatment adherence was assessed only among patients receiving disease-modifying therapy (DMT) (*n* = 310), and this subgroup was used for adherence-related analyses.

**Results:**

The mean age of participants was 43 years, and 58.3% were female. Disease severity was classified as mild in 50.4%, moderate in 34.7%, and severe in 15.0% of participants. Low physical and mental QoL were reported in 34.9% and 31.6%, respectively. Lower QoL was significantly associated with greater disease severity (β = −0.85, *p* = 0.001), illness duration >10 years (β = −0.35, *p* = 0.002), fatigue (β = −1.10, *p* < 0.001), anxiety (β = −1.00, *p* < 0.001), depression (β = −1.20, *p* < 0.001), cognitive impairment and treatment non-adherence (evaluated only among DMT-treated patients). Moderate-to-high physical activity (β = 0.60, *p* = 0.001) and strong social support (β = 0.70, *p* = 0.001) were associated with higher QoL.

**Conclusions:**

QoL among MS patients was strongly influenced by disease severity, treatment adherence (among patients receiving DMT), fatigue, psychological distress, cognitive function, physical activity and social support. Interventions targeting these modifiable factors may improve patient outcomes. This study provides important evidence from Ethiopia and helps address a significant knowledge gap regarding MS care in resource-limited settings.

## Introduction

Multiple sclerosis (MS) is a chronic, immune-mediated disorder of the central nervous system characterized by inflammation, demyelination and neurodegeneration, leading to diverse physical, cognitive and emotional impairments [1–3]. Globally, MS is a major contributor to long-term disability among young and middle-aged adults, affecting multiple functional domains and significantly reducing quality of life (QoL) [4,5]. Given the multifaceted and unpredictable nature of MS, understanding the determinants of QoL is essential for improving patient-centred care and guiding targeted interventions [6,7].

Previous studies have identified a range of clinical, psychological and behavioural factors influencing QoL in MS patients, including disease severity, duration of illness, fatigue, cognitive impairment, comorbid anxiety and depression, treatment adherence, levels of physical activity, and social support [7–13]. Regular exercise and strong social relationships, for example, have been associated with improved symptom management and better QoL outcomes [14,15].

However, most of these findings originate from high-income countries, and their applicability to resource-limited settings – where access to rehabilitation, mental health support and community-based care may be constrained – remains insufficiently examined. In such contexts, psychological well-being and social support may play an especially critical role in coping and functional adaptation due to limited clinical resources.

Historically, MS was considered rare in sub-Saharan Africa, with prevalence estimates of 1–5 per 100,000. However, emerging evidence challenges this assumption [16].

Increasing access to MRI and a growing number of neurologists have contributed to higher case detection in several African countries, including Ethiopia [17,18]. This suggests that MS may have been underdiagnosed rather than uncommon. A recent review highlighted the need for region-specific research to understand the lived experiences and disease-related challenges of MS patients in low-resource environments [18].

Despite these developments, there remains a lack of comprehensive, multicentre evidence on QoL among MS patients in Ethiopia. The interplay between clinical disability, psychological distress, treatment adherence, physical activity and social support in shaping QoL in the Ethiopian setting has not yet been fully characterized.

**Figure 1.**
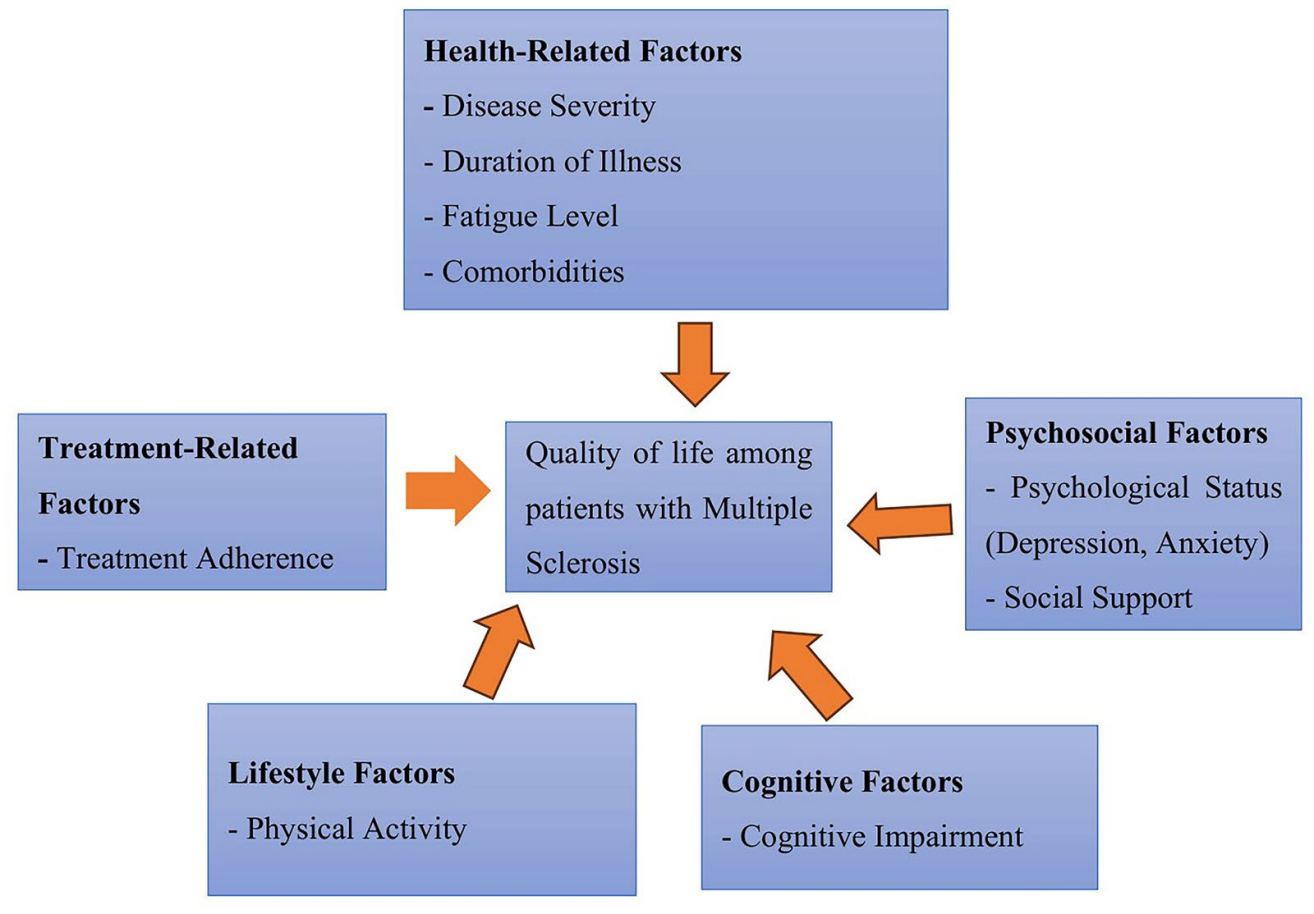
Conceptual framework of factors influencing quality of life among patients with multiple sclerosis.

To address this gap, the present study assessed factors influencing QoL among MS patients attending the University of Gondar Hospital, Debre Tabor Hospital and Felege Hiwot Hospital in Northwest Ethiopia. Figure 1 illustrates the conceptual framework guiding this study, depicting the hypothesized relationships between clinical characteristics, psychological and cognitive factors, behavioural factors (treatment adherence and physical activity), social support and QoL outcomes.

Therefore, this study aimed to identify clinical, psychological and social determinants of QoL among MS patients in Northwest Ethiopia (Figure 1).

## Methods

### Study design and setting

This six-month multicentre cross-sectional study was conducted from January to June 2024 across three public referral hospitals in Northwest Ethiopia: the University of Gondar, Debre Tabor and Felege Hiwot. These hospitals were selected due to their provision of specialized neurological services and higher caseloads of MS patients, making them representative of MS care in the region. Patients were recruited during scheduled neurology outpatient follow-up visits (e.g. routine monitoring or medication-refill appointments) as part of the study protocol separate from routine clinical care.

### Study population

The study population included MS patients aged 18 years or older with a confirmed diagnosis based on the 2017 McDonald criteria. Patients who had received follow-up care for at least six months and provided written informed consent were eligible. Patients were approached for participation when they attended these routine clinic visits, and their treatment status (receiving disease-modifying therapy (DMT) vs. untreated) at the time of assessment was recorded. Patients on any standard MS therapy were included, and for those on treatment the specific DMT and its initiation date were noted. Patients not receiving DMT remained eligible for the study because the primary objective was to assess QoL and its associated factors in the overall MS cohort. However, adherence assessment was applicable only to participants receiving DMT at the time of data collection. Cognitive status was initially screened using the Montreal Cognitive Assessment (MoCA); patients scoring <15 (indicating severe cognitive impairment) were excluded to ensure ability to provide reliable responses. Patients with major comorbid psychiatric disorders or acute medical conditions that could interfere with quality-of-life assessment were also excluded. These exclusions may introduce some selection bias towards patients with milder functional impairment; therefore, this limitation is acknowledged.

### Sample size and sampling techniques

The sample size was calculated using a single population proportion formula with a prevalence assumption of 50% due to limited prior local data: $\frac{({Z\alpha /2)}^{2}}{E^{2}}\times P(1-P)$, where:

- Proportion (*p*) = 0.50
- Confidence level (*Z*) = 1.96 (95%)
- Margin of error (*d*) = 0.05
- Non-response rate = 10%
  $$n=\frac{{\left(1.96\right)}^{2}}{{\left(0.05\right)}^{2}}\times 0.5(1-0.5)=384$$

Adjusting for a 10% non-response rate:

$$\frac{n}{1-\text{non}-\text{response rate})}$$

$$\text{nfinal}=\frac{384}{\left(1-0.1\right)}=427$$

**Figure 2.**
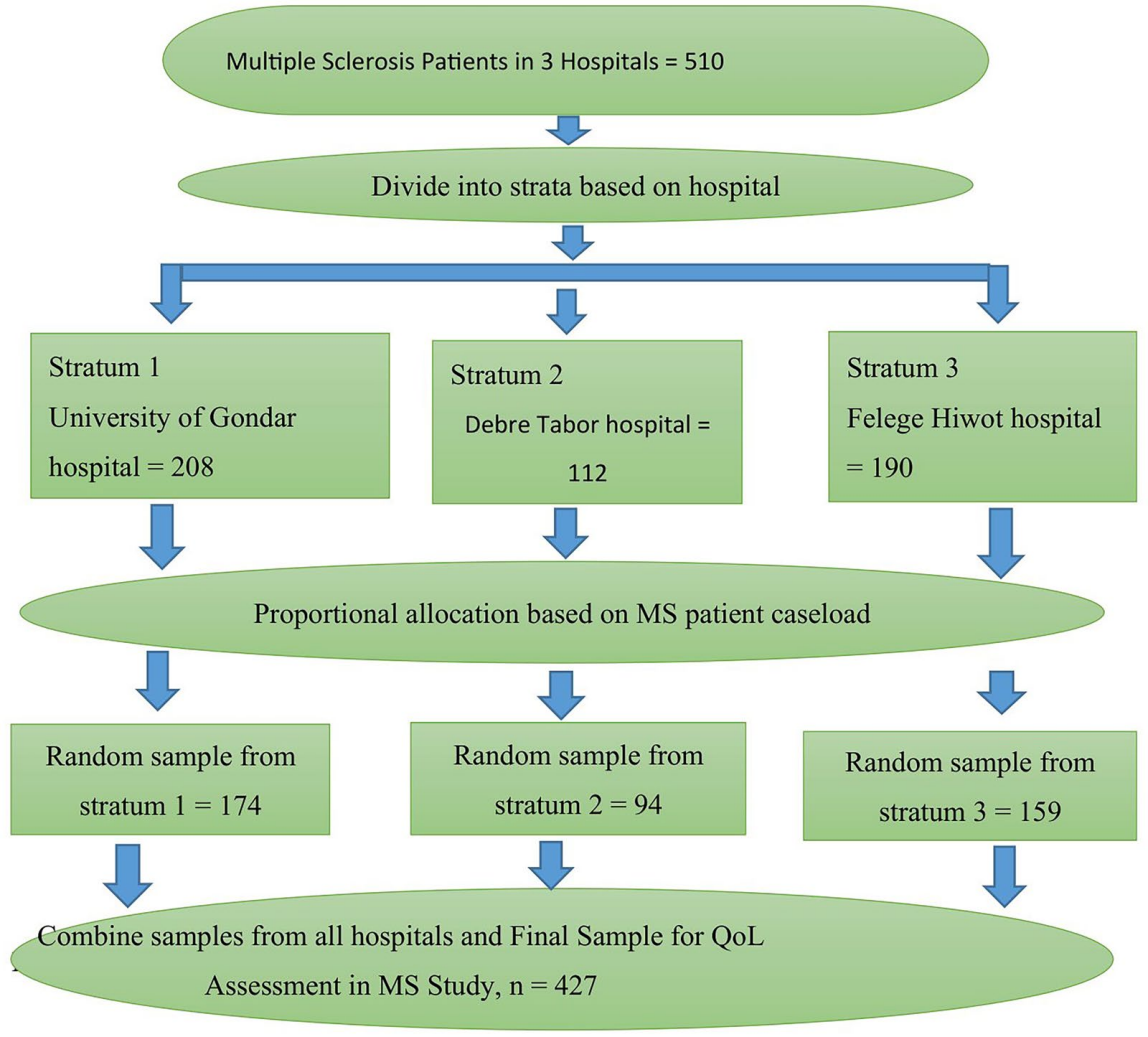
Sampling procedure for selection of study participants (*n* = 427).

A stratified random sampling technique allocated the sample proportionally across the three hospitals based on MS caseloads, followed by simple random sampling within each hospital (Figure 2).

### Rationale for stratified sampling

Stratification ensured proportional representation of patients across hospitals, improved precision, enhanced generalizability and controlled for facility-specific variations in patient characteristics and care practices.

### Data collection instruments

Data were collected using a structured questionnaire including sociodemographic, clinical, psychological, behavioural and social variables. Quality of life was assessed using the Multiple Sclerosis Quality of Life-54 (MSQOL-54) tool [19].

For regression analyses, the primary dependent variable was defined as the overall MSQOL-54 composite quality-of-life score, calculated from the physical health composite (PHC) and mental health composite (MHC) domains. Descriptive analyses additionally reported domain-specific physical and mental health QoL categories.

Disability severity was categorized using EDSS scores as mild (0–3.5), moderate (4.0–6.5) and severe (≥7.0), consistent with established classifications used in MS research [20]. Fatigue was assessed using the Fatigue Severity Scale (FSS). The FSS score was calculated as the mean score across the nine items, yielding a total score ranging from 1 to 7, with higher scores indicating greater fatigue severity [21]. Treatment adherence was assessed using the Medication Adherence Report Scale (MARS) [22] exclusively among participants who were receiving DMT at the time of assessment. Participants not receiving DMT were not administered the MARS questionnaire because adherence cannot be evaluated in the absence of prescribed therapy. Physical activity was assessed using the International Physical Activity Questionnaire (IPAQ) [23]; cognitive function using the MoCA [24]; anxiety and depression using the Hospital Anxiety and Depression Scale (HADS) [25]; and social support using the Multidimensional Scale of Perceived Social Support (MSPSS) [26].

Clinical variables included current MS treatments. We recorded any DMT used by participants, including common first-line agents such as interferon *β* − 1a/*β* − 1b, glatiramer acetate [20] and dimethyl fumarate, as well as newer therapies (e.g. fingolimod, teriflunomide, natalizumab and ocrelizumab). The start date of the current therapy was recorded to calculate treatment duration, and patients receiving no DMT were documented as untreated.

### Translation and pre-testing

Instruments not originally available in Amharic were translated and back-translated by bilingual experts. Cultural adaptation followed WHO guidelines [27]. A pilot study with 10% of the final sample was conducted to refine clarity. Internal consistency for all major scales was acceptable (Cronbach’s *α* ≥ 0.75).

### Data collection procedure

Questionnaires were administered through face-to-face interviews. To minimize interviewer bias, all data collectors underwent standardized training, were supervised throughout data collection, and routine inter-rater consistency checks were performed. Clinical data were extracted from patient records by trained clinicians. The extracted clinical data included details of MS treatment regimens (medication name and initiation date). These data were used to calculate each patient’s current treatment duration and classify DMT type. We also recorded the number of patients not on any DMT at the time of assessment. The MARS questionnaire was administered only to participants currently receiving DMT, and adherence data were collected exclusively for this subgroup.

### Classification of QoL categories

QoL scores were analysed as continuous variables for regression analysis. For descriptive presentation, low, moderate and high QoL categories were derived using quartile-based cut-points, consistent with previous MSQOL-54 applications in similar populations [19,20].

### Data analysis

Data were entered, cleaned and validated using EpiData, then exported to SPSS version 26 (SPSS Inc., Chicago, IL). Descriptive statistics summarized participant characteristics. Predictors of QoL were analysed using bivariate and multivariate linear regression. Variables with *p* < 0.20 in bivariate analysis were included in multivariate models. Linearity and normality of residuals were checked using Q–Q plots, and multicollinearity using variance inflation factors (VIFs < 5). Data analyses were performed using complete-case data. Participants with missing information on any variables included in the analyses were excluded using listwise deletion. Consequently, all analyses of QoL and its predictors were based on 427 participants with complete data. We included treatment-related variables in the regression models, testing whether DMT use (yes/no), DMT class (injectable vs. oral vs. infusion) or treatment duration predicted QoL. All adherence analyses, including estimation of adherence levels and comparisons of MARS scores across DMT categories, were restricted to the subgroup of participants receiving DMT. The untreated participants were excluded from adherence analyses but were retained in analyses describing the overall study cohort and quality-of-life outcomes, where treatment status (DMT use vs. untreated) was included as a clinical variable. Statistical significance was set at *p* < 0.05.

## Results

### Participant characteristics

A total of 427 participants with MS were enrolled, representing a diverse demographic profile. The majority were female (58%), and most were aged 31–45 years. Over half were married, and educational attainment varied, with a substantial proportion having secondary education or less. Employment status was mixed, with roughly 40% employed, one-third unemployed, and smaller fractions of students and retirees (Table 1).

**Table 1.** Sociodemographic characteristics of the study population (*n* = 427).

Variable	Category	*n* (%)
Age (years)	18–30	102 (24)
	31–45	145 (34)
	46–60	115 (27)
	>60	65 (15)
Gender	Male	178 (42)
	Female	249 (58)
Marital status	Single	112 (26)
	Married	228 (53)
	Divorced/widowed	87 (20)
Education	No formal/primary	203 (48)
	Secondary	138 (32)
	Diploma and above	86 (20)
Employment	Employed	168 (39)
	Unemployed	146 (34)
	Student/retired	113 (26)

### Clinical profile of MS patients

Participants demonstrated heterogeneity in disease severity, duration, fatigue, treatment status and adherence. Most retained mild to moderate disability (mean EDSS = 2.5–5.0), and the majority had been diagnosed for less than 10 years. Fatigue was common, with moderate or high levels reported by the majority.

Regarding immunomodulatory treatment, 223 (52%) participants were receiving injectable DMTs, 87 (21%) were receiving oral DMTs, and 117 (27%) were not receiving DMT at the time of assessment. Treatment adherence was assessed exclusively among the 310 participants receiving DMT. Among these treated patients, adherence was generally high, with 223 (72.0%) classified as adherent and 87 (28.0%) classified as non-adherent according to the MARS. Psychological distress was prevalent, with more than half of participants screening positive for anxiety and depression (Table 2).

**Table 2.** Clinical characteristics (overall cohort, *n* = 427; treatment adherence assessed among DMT-treated participants, *n* = 310).

Variable	Category	Mean (SD)/median (range)	*n* (%)
Disease severity (EDSS)	Mild (0–3.5)	2.5 (0–3.5)	215 (50)
	Moderate (4.0–6.5)	5.0 (4.0–6.5)	148 (35)
	Severe (7.0–9.5)	7.5 (7.0–9.5)	64 (15)
Duration of illness (years)	<5	3.0	185 (43)
	5–10	7.0	160 (38)
	>10	12.0	82 (19)
Treatment (disease-modifying therapy, DMT)	Injectable DMT (e.g. interferon β, glatiramer acetate)	–	223 (52)
	Oral DMT (e.g. fingolimod, teriflunomide, dimethyl fumarate)	–	87 (21)
	No DMT (untreated)	–	117 (27)
Treatment adherence (*n* = 310 DMT-treated participants only)	Adherent	75.4 (15.2)	223 (72.0)
	Non-adherent	45.6 (10.3)	87 (28.0)
Fatigue (FSS)	Low	2.2 (0.8)	119 (28)
	Moderate	4.0 (1.1)	182 (43)
	High	6.2 (1.3)	126 (29)

### Clinical profile of MS patients

Participants demonstrated heterogeneity in disease severity, duration, fatigue, treatment status and adherence. Most retained mild to moderate disability (mean EDSS = 2.5–5.0), and the majority had been diagnosed for less than 10 years. Fatigue was common, with moderate or high levels reported by the majority.

Regarding immunomodulatory treatment, 223 (52%) participants were receiving injectable DMTs, 87 (21%) were receiving oral DMTs, and 117 (27%) were not receiving DMT at the time of assessment. Treatment adherence was assessed exclusively among the 310 participants receiving DMT. Among these treated patients, adherence was generally high, with 223 (72.0%) classified as adherent and 87 (28.0%) classified as non-adherent according to the MARS. Psychological distress was prevalent, with more than half of participants screening positive for anxiety and depression (Table 2).

### Psychological and cognitive assessment

Clinically significant anxiety and depression were reported by over half of the participants, indicating a substantial mental health burden. Cognitive impairment was common, with nearly two-thirds showing some level of impairment on the MoCA (Table 3).

**Table 3.** Psychological and cognitive characteristics (*n* = 427).

Variable	Category	Mean (SD)	*n* (%)
Anxiety (HADS-A)	Normal	5.2 (1.8)	190 (45)
	Mild–severe	–	237 (55)
Depression (HADS-D)	Normal	4.7 (2.1)	178 (42)
	Mild–severe	–	249 (58)
Cognitive function (MoCA)	Normal	27.5 (2.5)	160 (38)
	Mild–severe	–	267 (62)

### Quality of life assessment

Quality of life scores revealed that most participants reported moderate physical and mental QoL, with roughly one-third experiencing low QoL in each domain.

For clarity, ‘Physical Health’ and ‘Mental Health’ represent the MSQOL-54 PHC and MHC scores, respectively. Categories (low, moderate, high) were derived using quartile-based cut-points for descriptive interpretation, with higher scores reflecting better perceived QoL.

These results highlight the functional and psychological impact of MS in this cohort (Table 4).

**Table 4.** Quality of life scores measured by MSQOL-54.

Domain	MSQOL-54 subscale definition	Category	Mean (SD)	*n* (%)
Physical health	PHC: physical functioning, role limitation, pain, energy and health perception	Low	39.8 (10.2)	149 (35.0)
		Moderate	58.6 (8.4)	206 (48.0)
		High	76.4 (6.9)	72 (17.0)
Mental health	MHC: emotional well-being, cognitive function, emotional role limitation and social functioning	Low	41.2 (11.1)	135 (31.6)
		Moderate	62.7 (8.3)	204 (47.8)
		High	80.2 (7.1)	88 (20.6)

MSQOL-54: Multiple Sclerosis Quality of Life-54; PHC: physical health composite; MHC: mental health composite.

Higher scores indicate better quality of life. Categories were derived using quartile-based cut-off points.

### Lifestyle and social support

Physical activity levels were generally low to moderate, while social support was moderate for most participants. However, a substantial minority lacked strong social networks, which may negatively affect coping and overall QoL (Table 5).

**Table 5.** Lifestyle and social support variables (*n* = 427).

Variable	Category	Mean (SD)	*n* (%)
Physical activity (IPAQ)	Low	28.5 (7.6)	170 (40)
	Moderate	45.2 (9.3)	185 (43)
	High	62.5 (8.7)	72 (17)
Social support (MSPSS)	Low	35.7 (11.8)	122 (29)
	Moderate	55.0 (9.2)	215 (50)
	High	70.3 (10.5)	90 (21)

### Predictor variables influencing quality of life: bivariate regression analysis

The bivariate analysis examined the associations between clinical and psychosocial variables and overall QoL (overall MSQOL-54 composite score, derived from physical and mental health domains) in patients with MS.

Disease severity was negatively associated with overall MSQOL-54 scores, indicating that individuals with greater disability reported poorer functioning and well-being (*p* < 0.01). Similarly, a longer duration of illness was associated with lower overall QoL scores, particularly among individuals living with MS for >10 years (*p* < 0.05).

In contrast, treatment adherence and higher physical activity levels showed positive associations with overall MSQOL-54 scores (*p* < 0.01). Higher levels of fatigue, anxiety and depression were all significantly associated with lower overall QoL (*p* < 0.01). Cognitive function (MoCA scores) showed a positive correlation with overall MSQOL-54 scores (*p* < 0.01), while perceived social support was also positively associated with QoL (*p* < 0.01) (Table 6).

**Table 6.** Bivariate associations between lifestyle/social factors and quality of life in MS patients.

Predictor variable	QoL outcome (MSQOL-54 domain)	Statistical test	*p* Value
Physical activity (IPAQ)	Physical health QoL	ANOVA	0.022
Physical activity (IPAQ)	Mental health QoL	ANOVA	0.018
Social support (MSPSS)	Physical health QoL	Spearman’s correlation	0.006
Social support (MSPSS)	Mental health QoL	Spearman’s correlation	0.005

*p* < 0.05 was statistically significant.

### Impact of clinical and psychosocial factors on overall quality of life in patients with multiple sclerosis: multiple regression analysis

Multiple regression analysis examined the independent effects of clinical, psychological and lifestyle factors on overall QoL (MSQOL-54 composite score) among patients with MS. The reported *β* values represent adjusted unstandardized regression coefficients (*B*) from the final multivariable linear regression model.

The final adjusted model demonstrated that increasing disease severity was strongly associated with reduced overall MSQOL-54 scores. Compared with patients with mild disability (EDSS 0–3.5), those with moderate disability (EDSS 4–6.5) showed significantly lower QoL (*β* = −0.25, 95% CI: −0.48 to −0.02, *p* = 0.035), while severe disability (EDSS >7) showed a markedly greater reduction (*β* = −0.85, 95% CI: −1.28 to −0.42, *p* = 0.001).

Duration of illness showed a time-dependent effect. Patients with MS duration >10 years had significantly lower overall MSQOL-54 scores (*β* = −0.35, *p* = 0.002).

Treatment adherence emerged as a strong protective factor, with non-adherent patients reporting significantly poorer overall QoL (*β* = −0.95, *p* < 0.001).

Fatigue demonstrated a dose–response association with poorer QoL. Similarly, anxiety and depression each demonstrated progressive reductions in overall MSQOL-54 scores across symptom severity levels.

Cognitive impairment remained independently associated with reduced QoL after adjustment for other clinical and psychosocial variables. Compared with cognitively normal participants, mild impairment (*β* = −0.35, *p* = 0.012), moderate impairment (*β* = −0.60, *p* = 0.004) and severe impairment (*β* = −1.00, *p* = 0.001) demonstrated progressively lower MSQOL-54 scores, suggesting a dose–response relationship between worsening cognitive status and poorer QoL. Conversely, physical activity and social support demonstrated protective effects (Table 7).

**Table 7.** Multivariable linear regression analysis of factors associated with quality of life among multiple sclerosis patients in northwest Ethiopia.

Predictor variable	Category	*β* (beta)	Std. error	*p* Value	95% CI
Disease severity (EDSS)	Mild (0–3.5)	Ref.	–	–	–
	Moderate (4–6.5)	−0.25	0.12	**0.035**	**(−0.48, −0.02)**
	Severe (>7)	−0.85	0.22	**0.001**	**(−1.28, −0.42)**
Duration of illness	<5 years	Ref.	–	–	–
	5–10 years	−0.12	0.10	0.18	(−0.32, 0.08)
	>10 years	−0.35	0.11	**0.002**	**(−0.56, −0.14)**
Treatment adherence	Adherent	Ref.	–	–	–
	Non-adherent	−0.95	0.18	**<0.001**	**(−1.30, −0.60)**
Fatigue (FSS)	Low	Ref.	–	–	–
	Moderate	−0.40	0.15	**0.008**	**(−0.69, −0.11)**
	High	−1.10	0.22	**<0.001**	(−1.53, −0.67)
Anxiety (HADS-A)	Normal	Ref.	–	–	–
	Mild	−0.30	0.14	**0.025**	**(−0.58, −0.02)**
	Moderate	−0.65	0.20	**0.001**	**(−1.03, −0.27)**
	Severe	−1.00	0.25	**<0.001**	**(−1.48, −0.52)**
Depression (HADS-D)	Normal	Ref.	–	–	–
	Mild	−0.45	0.12	**0.005**	**(−0.69, −0.21)**
	Moderate	−0.70	0.17	**0.002**	**(−1.02, −0.38)**
	Severe	−1.20	0.22	**<0.001**	**(−1.63, −0.77)**
Cognitive function (MoCA)	Normal	Ref.	–	–	–
	Mild Impairment	−0.35	0.10	**0.012**	**(−0.55, −0.15)**
	Moderate Impairment	−0.60	0.20	**0.004**	**(−0.98, −0.22)**
	Severe Impairment	−1.00	0.22	**0.001**	**(−1.44, −0.56)**
Physical activity (IPAQ)	Low	Ref.	–	–	–
	Moderate	0.25	0.12	**0.030**	**(0.02, 0.48)**
	High	0.60	0.18	**0.001**	**(0.24, 0.96)**
Social support (MSPSS)	Low	Ref.	–	–	–
	Moderate	0.35	0.10	**0.012**	**(0.15, 0.55)**
	High	0.70	0.22	**0.001**	**(0.26, 1.14)**

*p <* 0.05 was statistically significant.

Model diagnostic procedures demonstrated acceptable model assumptions. Residual normality and linearity were assessed using Q–Q plots and residual-versus-fitted plots, showing no substantial deviations from assumptions. Multicollinearity diagnostics indicated acceptable independence among predictors, with VIF values ranging from 1.24 to 3.41, below the commonly accepted threshold of 5.

## Discussion

This study investigated the clinical and psychosocial factors influencing QoL among patients with MS in Ethiopia. The findings demonstrate that disease severity, fatigue, anxiety, depression, cognitive impairment and treatment non-adherence are significant predictors of reduced QoL, while higher physical activity and stronger social support are associated with improved QoL. These results highlight the multidimensional nature of QoL in MS and the importance of addressing both physical and psychological components of care.

Disease severity emerged as a strong determinant of QoL. Patients with moderate and severe disability experienced significant declines in QoL compared to those with milder disease. This aligns with previous research indicating that higher EDSS scores are consistently associated with functional limitations, reduced mobility and greater dependence, which are associated with lower QoL [28]. Similarly, the negative impact of illness duration beyond 10 years supports earlier findings that long-term disease burden is associated with cumulative physical and social challenges [26].

Fatigue showed one of the strongest effects on reduced QoL, consistent with prior studies identifying fatigue as one of the most disabling symptoms of MS [29]. In our Ethiopian cohort, the exceptionally high prevalence of fatigue may reflect the lack of specialized rehabilitation and symptom-management services. In many parts of Africa, formal fatigue-management programs (such as energy-conservation education, structured exercise regimens or pharmacologic therapy) are scarce. Interventions like patient education on pacing techniques, access to physical therapy, and psychosocial support (e.g. cognitive-behavioural therapy for fatigue) have been shown elsewhere to reduce fatigue and improve QoL. The absence of these resources locally could partly explain the burden of fatigue observed in our sample.

Anxiety and depression also demonstrated clear dose–response effects on QoL, underscoring the emotional burden of MS and supporting the need for integrated mental health assessment in routine MS care [12,30–32]. The high prevalence of mood symptoms in this sample further reinforces the unmet mental health needs in resource-limited settings, where access to psychiatric services remains limited [33]. Given the cross-sectional nature of this study, the direction of these relationships cannot be determined; poorer QoL may contribute to psychological distress, psychological distress may contribute to poorer QoL, or both may occur simultaneously. Given that MS-related mood symptoms worsen with stress and isolation, strengthening social support (as our data suggest) and providing counselling could partially mitigate their impact.

Although cognitive impairment was associated with lower QoL in the unadjusted analyses, it was not retained as an independent predictor in the final adjusted regression model. This suggests that the observed relationship may be partly explained by other clinical and psychosocial factors included in the model. Nevertheless, cognitive impairment remains clinically relevant, as previous studies have shown that cognitive difficulties can adversely affect independence, work productivity and social participation [34]. In the Ethiopian context, where cognitive rehabilitation programs are scarce, routine cognitive screening and counselling (e.g. memory strategies, occupational therapy referrals) become particularly important to help maintain function and QoL.

Treatment non-adherence was assessed exclusively among patients receiving DMT and was therefore restricted to this subgroup of treated participants. Within this subgroup, non-adherence was strongly associated with poorer QoL. This finding is consistent with previous studies showing that non-adherent patients experience more relapses and accelerated disability progression [35,36]. Indeed, numerous studies have demonstrated that better adherence to DMTs is linked to improved clinical outcomes: adherent patients have fewer relapses, slower disability accumulation and even better psychosocial functioning [37,38]. For example, Kołtuniuk et al. found that in MS, adherence supports emotional stability and cognitive function, with a clear positive correlation to overall QoL [39]. Conversely, non-adherent patients often report worse symptom control and more mood disturbances, which may contribute to lower QoL. However, because of the cross-sectional design, reverse causality cannot be excluded; patients with poorer QoL may also face greater challenges adhering to treatment. These findings therefore apply specifically to patients receiving DMT and should not be generalized to untreated individuals.

Contextual factors in Ethiopia likely exacerbate adherence challenges. Many first-line DMTs are either unavailable or unaffordable for patients in low-resource settings. A recent survey of MS care in Africa noted that essential DMTs like interferon-beta and glatiramer acetate are only available in a minority of countries [40]. In Ethiopia specifically, most patients rely on older therapies (or intermittently available medications) because newer oral and infusion DMTs are largely out of reach. This limited treatment availability means that even if patients wish to adhere, their options are constrained. Strengthening medication counselling and addressing affordability or supply barriers are therefore critical to ensure patients can remain on effective therapies.

Conversely, physical activity and social support were positively associated with QoL. Engaging in regular physical activity may be related to better physical function, lower fatigue levels and improved mood, as described in prior evidence advocating exercise as an important component of MS management [41,42]. However, it is equally plausible that individuals with better QoL are more able to participate in physical activity. In settings like ours, even simple home-based exercise programs or community activity groups may provide meaningful support for patients with MS.

Similarly, social support was positively associated with QoL. Social support may help individuals cope with emotional stress and reduce feelings of isolation, particularly in collective cultural settings where family networks are central [43,44]. These findings are consistent with research from other African contexts, which similarly highlight the importance of social support systems and community integration in chronic illness coping. In many parts of Ethiopia, formal rehabilitation services are limited, so informal networks of family and friends often serve as the primary source of support. Given the cross-sectional design, it is also possible that individuals with better QoL maintain stronger social relationships. Promoting community-based support groups or patient associations may further strengthen these existing networks.

Taken together, these results underscore the need for a holistic approach to MS care in Ethiopia. Alongside routine medical management, clinicians should be aware of the high burden of fatigue and mood symptoms and proactively offer education and symptomatic therapies. The observed associations between treatment adherence (among DMT-treated patients), physical activity, social support and QoL suggest that these factors may represent potentially modifiable targets for intervention, although longitudinal studies are needed to clarify the direction and causality of these relationships. Enhancing support services, including low-cost community-based interventions, may help address some of the factors associated with poorer QoL identified in this study.

### Implications for practice

These findings suggest that improving QoL among individuals with MS requires a holistic approach. Health systems should integrate:

- Routine mental health screening and counselling services.
- Structured fatigue management and energy-conservation training.
- Community-based physical activity and rehabilitation programs.
- Family and peer support interventions to strengthen social networks.
- Adherence support strategies, including medication counselling and financial assistance.

Such interventions may be particularly important in low-resource settings where access to specialist neurological care is limited.

### Study limitations

This study has several limitations. Its cross-sectional design limits causal inference, and reliance on self-reported data introduces potential recall and social desirability bias. Additionally, the IPAQ may overestimate physical activity levels, which should be interpreted cautiously. Some measures, such as the fatigue and depression scales, have not been formally validated in Ethiopian cultural contexts, which may influence symptom interpretation.

Although translation and back-translation procedures were conducted according to recommended guidelines and all major instruments demonstrated acceptable internal consistency (Cronbach’s *α* ≥ 0.75), several standardized assessment tools used in this study (including FSS, MARS, IPAQ, MSPSS and selected MSQOL-54 components) have not been formally psychometrically validated in Ethiopian MS populations. Therefore, cultural and contextual differences may have affected the interpretation of certain constructs, potentially influencing measurement accuracy and comparability with findings from other settings.

Recruitment from tertiary hospitals may also limit generalizability to community or rural populations. Finally, individuals with severe cognitive impairment were excluded, potentially underestimating the impact of cognitive dysfunction on QoL.

## Conclusions

This multicentre study identifies both clinical and psychosocial determinants of QoL among Ethiopian MS patients, with disease severity, fatigue, depression, anxiety and cognitive impairment serving as key negative predictors, and physical activity and social support acting as protective factors. These findings highlight the need to integrate mental health care, rehabilitation services, adherence support and community-based social interventions into MS management strategies in Ethiopia. As recognition of MS increases in sub-Saharan Africa, these results provide timely evidence to guide national clinical guidelines and health policy development.

## Data Availability

The datasets supporting the findings of this study are available from the corresponding author upon reasonable request. Requests for access to the data should be directed to the corresponding author, who will assess and, where appropriate, facilitate access in accordance with ethical, privacy and any other applicable restrictions. Any data that cannot be shared publicly are subject to confidentiality, ethical or privacy considerations. No datasets have been deposited in a public repository.
